# A genome-wide synthetic dosage lethality screen reveals multiple pathways that require the functioning of ubiquitin-binding proteins Rad23 and Dsk2

**DOI:** 10.1186/1741-7007-7-75

**Published:** 2009-11-12

**Authors:** Chang Liu, Dewald van Dyk, Yue Li, Brenda Andrews, Hai Rao

**Affiliations:** 1 Institute of Biotechnology, Department of Molecular Medicine, University of Texas Health Science Center at San Antonio, San Antonio, Texas 78245, USA; 2 Banting & Best Department of Medical Research, Department of Molecular & Medical Genetics, University of Toronto, Toronto, Ontario, Canada

## Abstract

**Background:**

Ubiquitin regulates a myriad of important cellular processes through covalent attachment to its substrates. A classic role for ubiquitin is to flag proteins for destruction by the proteasome. Recent studies indicate that ubiquitin-binding proteins (e.g. Rad23, Dsk2, Rpn10) play a pivotal role in transferring ubiquitylated proteins to the proteasome. However, the specific role of these ubiquitin receptors remains poorly defined. A key to unraveling the functions of these ubiquitin receptors is to identify their cellular substrates and biological circuits they are involved in. Although many strategies have been developed for substrate isolation, the identification of physiological targets of proteolytic pathways has proven to be quite challenging.

**Results:**

Using a genome-wide functional screen, we have identified 11 yeast genes that cause slower growth upon their overexpression in cells lacking two ubiquitin-binding proteins Rad23 and Dsk2. Our results suggest that proper functioning of Rad23 and Dsk2 is required for efficient pheromone response, transcription, amino acid metabolism, and DNA damage response. Two proteins identified by the screen are shown to be proteolytic substrates of Dsk2, validating the large scale synthetic dosage lethality screen as a new strategy for identifying substrates of a specific degradation pathway.

**Conclusion:**

In conclusion, as proof-of-concept, we show that a synthetic dosage lethality screen, which is based on the toxicity induced by gene overexpression, offers an effective, complementary method to elucidating biological functions of proteolytic pathways.

## Background

In eukaryotes, the 26S proteasome handles the majority of regulated proteolysis and is pivotal for the proper function of the cell [1,2]. Most proteins that are targeted to the proteasome for degradation are first modified by the ubiquitin (Ub) system [3]. Specifically, successive Ub molecules join to form a Ub chain on the substrates. How the ubiquitylated substrate is then delivered to and degraded by the 26S proteasome remains elusive [2,4-6]. It is now widely accepted that Ub-binding proteins (e.g. Rad23, Rpn10, and Cdc48) play important roles in facilitating substrate transfer to the proteasome. These proteins have distinct substrate specificity and functionally cooperate for substrate proteolysis [7-9]. However, how any one of these proteins works *in vivo *remains largely unknown [2,6].

Rad23 belongs to a family of proteins that contain both the Ub-associated (UBA) domain and a Ub-like (UBL) motif [2,4-6]. The UBA motif binds specifically to Ub chain/conjugates *in vivo *and *in vitro *[10-12]. The UBL motif directly binds the proteasome [13,14]. Through these domain-mediated interactions, UBA/UBL proteins link ubiquitylated proteins to the proteasome. In the budding yeast *Saccharomyces cerevisiae*, there are three UBA/UBL-containing proteins, Rad23, Dsk2 and Ddi1, which have been implicated in Ub-mediated proteolysis. The loss of *S. cerevisiae *Rad23 and Dsk2 leads to the stabilization of an artificially designed Ub-fusion substrate Ub^V76^-V-βgal [12,15], the cell cycle inhibitor Far1 [7], misfolded protein CPY* [16], an ER protein Hmg2 [8], and the homologues of Rad23 are involved in the degradation of the CDK inhibitor Rum1 [11], Pax3 [17] and the tumor suppressor p53 [18]. Consistent with the role of Rad23 and Dsk2 in post-ubiquitylation events, the stabilized substrates in *rad23Δ dsk2Δ *mutant cells are fully ubiquitylated. Rad23 and Dsk2 play overlapping functions since cells lacking both *RAD23 *and *DSK2 *are more sensitive to various stress conditions (e.g., higher temperature, canavanine, high salt) than either single mutant [19,20]. However, the biological pathways that require the functioning of Rad23 and Dsk2 remain to be identified.

Not all proteolytic substrates are degraded by the Rad23/Dsk2-dependent pathway. Other Ub-binding proteins, Rpn10, Rpn13, and the Cdc48-Ufd1-Npl4 complex also promote proteasome-mediated proteolysis and exhibit distinct substrate specificity [5,7-9,21]. For example, the cell cycle protein Clb2 requires Rpn10, but not Rad23 for its degradation. And Deg1-GFP is degraded by the Cdc48/Ufd1 pathway, independent of Rpn10 and Rad23. The principle underlying the division of labor among these Ub-binding proteins is far from clear. A key to unraveling the functions of these Ub receptors (e.g. Rad23, Rpn10) is to identify their cellular substrates and biological functions. For example, using mass spectrometry, more than 50 proteins were shown to be preferentially accumulated in cells lacking *RPN10 *[22,23]. These putative Rpn10 substrates are involved in cell cycle control, protein transport, metabolism, and transcription.

Here, we employ a new method to identify the biological function of Rad23 and Dsk2. The lack of overt phenotypes in *rad23Δ dsk2Δ *mutant cells likely reflects that redundant pathways are employed in buffering against mutations. Using a synthetic enhancement approach, we have isolated 11 genes that when overexpressed cause slow growth in *rad23Δ dsk2Δ *mutants. Our data expand the physiological processes that involve Rad23 and Dsk2. Furthermore, we show that two of the encoded proteins isolated by this method are ubiquitylated and degraded by the Dsk2-dependent pathway. Our results also suggest that the synthetic dosage lethality screen can be used to isolate physiological targets of a specific proteolytic pathway.

## Methods

### Strains, media and plasmids

Yeast cultures were grown in rich (YPD) or synthetic media containing standard ingredients and 2% glucose (SD medium), 2% raffinose (SR medium), or 2% raffinose + 2% galactose (SRG medium). The synthetic genetic array (SGA) compatible S288c strain Y8835 (*MAT ura3::natR can1::STE2pr-Sp_his5 lyp1 his3 1 leu2 0 met15 0*) and isogenic *rad23Δ dsk2Δ *(*rad23::kanMX dsk2::natR*) cells were used for all assays involving the deletions of *RAD23 *and *DSK2*. Strains JD52 (*Mat****a ****lys2 ura3 trp1 his3 leu2*), JD55 (*UBR1::HIS3 *in JD52 background) and *ufd1-1 *were obtained from Dr. Alex Varshavsky (California Institute of Technology) [24]. Haploid strains bearing *rad23Δ*, *dsk2Δ*, *ddi1Δ, rpn10Δ, rpn13Δ, kre22Δ*, in the BY4741 background were obtained from Open Biosystems (Huntsville, AL, USA).

For the Synthetic Dosage Lethality (SDL) screens, haploids were selected on synthetic dextrose medium (2% glucose; 1.7 g/L yeast nitrogen base w/o ammonium sulfate and amino acids; 1 g/L monosodium glutamic acid; 2 g/L amino acid dropout mix lacking uracil, arginine, lysine and histidine), supplemented with the following antibiotics 100 mg/L clonNAT (Werner BioAgents Jena Germany), 200 mg/L geneticin (Invitrogen, Carlsbad, CA, USA), 50 mg/L L-canavanine (Sigma, St. Louis, MO, USA) and 50 mg/L S-(2-Aminoethyl)-L-cysteine hydrochloride (Sigma).

The plasmids expressing the proteasomal substrates Arg-βgal, Scc1, or Ub^V76^-V-βgal, Ricin A chain (RTA) have been previously described [24-26] and substrate expression are regulated by the *GAL1 *promoter. The pEGH plasmids, which contain an individual yeast gene tagged with both Glutathione S-transferase (GST) protein and His6 at its amino-terminus, were previously described [27,28]. The movable ORF (MORF) clones were purchased from Open Biosystems (Huntsville, AL, USA). The plasmid expressing Ha-Ub was obtained from Dr. Mary Ann Osley (University of New Mexico, Albuquerque, NM, USA).

### Genome-wide synthetic dosage lethal screen

The *URA3*-marked overexpression library and the screening procedures used in this study were previously described [28]. Briefly, two isogenic synthetic genetic array (SGA)-compatible strains, Y8835 and *rad23Δ dsk2Δ*, were mated to an ordered yeast array expressing 5200 unique galactose-inducible genes. The arrayed strains were subjected to diploid selection, sporulation and two rounds of haploid selection [28-30], to give rise to an output array of duplicated colonies carrying the desirable natR-marked deletion and one unique galactose-inducible gene. The haploid arrays were finally replica-pinned onto media containing 2% glucose (uninduced condition) or 2% galactose (induced gene expression condition). The colonies on the glucose and galactose plates were photographed after two and three days of incubation, respectively. Overexpressed genes that uniquely caused a reduction in colony size of more than 20% in the *rad23Δ dsk2Δ *background were considered for downstream analysis.

### Expression shut-off assay and proteasome inhibition treatment

Yeast cells carrying plasmids expressing hexahistidine (His6) and GST double-tagged proteins from the *GAL1 *promoter were grown at 30°C to an OD_600 _of approximately 1 in SR-ura medium with auxotrophic supplements and 2% raffinose as the carbon source. Protein expression was induced with 2% galactose for 3 h and then repressed by the addition of 2% glucose. Cycloheximide (100 μg/ml) was also added to stop translation. Samples were withdrawn at the indicated time points and harvested by centrifugation. Proteins were extracted by glass bead lysis of cells, processed for immunoprecipitation with Glutathione sepharose beads (GE-Healthcare, Piscataway, NJ, USA), and resolved by 10% SDS-PAGE. Immunoblots were probed with anti-His6 antibody (Abcam, Cambridge, UK) followed by detection with goat anti-mouse Horseradish Peroxidase (HRP) conjugate using Chemiluminescent reagents (GE-Healthcare). The stable protein Rpt5 was used as a loading control in the expression shutoff experiments.

Proteasome inhibition was performed as described [31]. Briefly, yeast cells were grown in synthetic media using proline as the only nitrogen source. SDS (0.003%) was added to the media 3 h before galactose induction. MG132 (75 μM; Biomol) was added 30 min before the addition of glucose. Samples were collected at indicated time points and processed as described above.

### Detection of ubiquitylated substrates

Yeast cells expressing GST-tagged substrates and Ha-tagged Ub or myc-tagged Ub were grown in galactose-containing SG medium to an OD_600 _of approximately 1. Cells were lysed with glass beads and immunoprecipitated with Glutathione sepharose beads for 2 h at 4°C. The immunoprecipitates were resolved by SDS-PAGE, transferred to Polyvinylidene fluoride (PVDF) membrane, and immunoblotted with anti-Ha antibody (Covance, Berkeley, CA, USA), followed by anti-mouse HRP conjugates and ECL reagents.

## Results

### Accumulation of proteasomal substrates in degradation mutants can lead to growth retardation

We set out to look for physiological substrates of Rad23 and Dsk2. Many strategies have been developed in the past to isolate the substrates of a particular Ub/proteasome pathway with varying degrees of success. Most of these methods explore the facts that substrates directly bind to the degradation machinery and/or substrates are ubiquitylated. Two challenges often encountered in these endeavors are the transient nature of the interaction between substrates and degradation components, and low levels of substrate expression *in vivo*.

**Figure 1:**
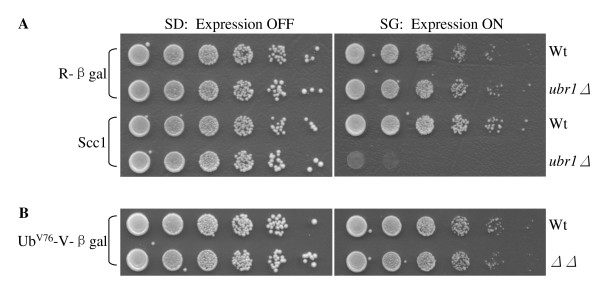
**Accumulation of some proteasomal substrates leads to slower cell growth**. **(A) **Compromised degradation of Scc1, but not Arg-βgal, in *ubr1Δ *mutant cells causes toxicity. The plasmids bearing the N-end rule substrate Arg-βgal or Scc1 were separately introduced into wild-type JD52 or *ubr1Δ *cells as indicated. The expression of these substrates are regulated by the galactose-inducible *GAL1 *promoter. Exponentially growing yeast cells were spotted onto glucose-containing SD-ura (expression off) and galactose-containing SG-ura (expression on) media in serial five-fold dilutions. The plates were incubated at 30°C for two to four days. The substrates used are labeled on the left of the panel, and the strains are labeled on the right. **(B) **Increased levels of the UFD substrate Ub^V76^-Val-βgal in *rad23 dsk2 *mutant cells do not affect cell growth. The spotting experiments were performed as in *A*.

To identify the physiological functions of Rad23 and Dsk2, we employed a synthetic dosage lethality screen [32,33]. The premise of SDL is that increased levels of a protein have little toxic effect on the growth of a wild-type strain but may cause growth retardation or even lethality in a mutant strain. Previous studies suggest that this strategy can be adapted to isolate substrates of the Ub/proteasome system. For example, overexpression of several cell cycle proteins (e.g., Clb2, Sic1, Scc1) in proteolysis-deficient cells (e.g. *apc *mutant, *cdc34 *mutant, *ubr1 *mutant) led to extremely slow cell growth (Figure 1A and references [24,34,35]). The growth defect induced by substrate Scc1 overexpression is particularly striking in the case of *ubr1Δ *cells lacking the E3 enzyme of the N-end rule degradation pathway, since the absence of *UBR1 *alone does not significantly alter cell growth [36].

We then wondered whether the toxicity might be related to the physiological functions of the substrates. In other words, would accumulation of large amounts of any substrate lead to growth retardation in proteolysis-deficient cells? We used an artificially designed N-end rule substrate Arg-βgal, which contains βgalactosidase from *E. coli *fused to a destabilizing residue Arg [37]. Deletion of *UBR1 *leads to the accumulation of Arg-βgal, which does not affect cell growth (Figure 1A). Similarly, we found that compromised degradation of another model substrate of the proteasome Ub^V76^-Val-βgal does not elicit toxicity in *rad23Δ dsk2Δ *mutants (Figure 1B) [12], indicating that the toxicity triggered by substrate accumulation may depend on the physiological function of the target protein. These results further suggest that the SDL screen can identify a subset of substrates of a given proteolytic pathway.

### Identification of yeast genes which cause slower growth in rad23 dsk2 mutant cells upon overexpression

**Figure 2:**
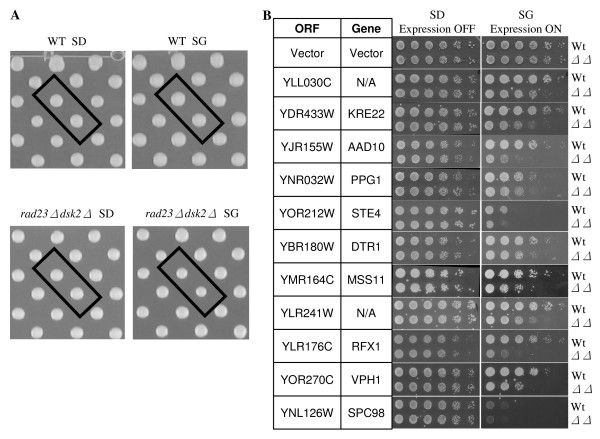
**Identification of yeast genes that cause stronger growth retardation in *rad23 dsk2 *mutant cells than in wild-type cells**. **(A) **Representative images from plasmid overexpression screen. Each of approximately 5280 yeast ORFs regulated by a galactose inducible Gal1 promoter was transformed separately into wild-type Y8835 and *rad23Δ dsk2Δ *mutant cells. Transformants were grown on media containing either glucose (SD, expression off) or galactose (SG, expression on). Each ORF is represented by two spots on the plate to reduce false positives. After two days for SD plates and three days for SG plates, colony sizes were scored for possible hits. Boxed spots are *YLL030C *on each plate. Strains and growth conditions are labeled at the bottom of each image. **(B) **A spotting assay indicates 11 genes cause preferential slow growth in *rad23Δ dsk2Δ *mutant cells. The spotting assay was conducted as described in Figure 1A. The identities of these genes and the corresponding strains are listed to the left and right of the panels, respectively.

The genome-wide SDL screen was carried out as previously described [28]. More than 5000 yeast genes fused to both glutathione S-transferase and His6 tags were separately introduced into wild-type or *rad23Δ dsk2Δ *mutant cells [28,29]. We compared the growth of wild type vs. *rad23Δ dsk2Δ *cells that expressed each of these yeast genes from a strong, regulatable *GAL1 *promoter. The expression of these genes was repressed in glucose containing media, but induced in galactose-containing media. Haploids carrying these plasmids were pinned onto both SD (glucose media) and SG (galactose media) plates. Following incubation for two to three days at 30°C (Figure 2A), the colony sizes of these plates were scored using a program developed in Dr. Boone's Lab (University of Toronto, Ontario, Canada). Upon their overexpression, a total of 41 genes were found to preferentially cause slower growth in *rad23Δ dsk2Δ *mutant cells. Sic1 and Far1, two known substrates of Rad23 and Dsk2 [7], were not isolated as positives in our screen because their overexpression leads to severe toxicity even in wild-type cells (data not shown), suggesting that genes, which severely reduce growth rate in wild-type cells may not be recovered in this type of SDL screen.

As is common in nearly all screens, we expected our approach to yield false positives, especially since a low cut-off (20% difference in colony size) is used to define a hit. The 20% cut-off ensures that most of the toxic genes are efficiently captured in our screens (Figure 2A). To eliminate false hits, we performed a serial spotting assay with each candidate identified. More specifically, wild-type or *rad23Δ dsk2Δ *mutant cells containing the candidate open reading frame (ORF) regulated by the *GAL1 *promoter were grown in non-inducible raffinose media to a similar OD_600_, and then spotted onto SD and SG plates in five-fold serial dilutions. The growth rates of wild-type and *rad23Δ dsk2Δ *mutant cells bearing a vector plasmid are similar under the condition (Figure 2B). Of the 41 genes identified in the primary screen, eleven genes were confirmed to cause noticeably slower growth in mutant cells than in wild-type cells upon overexpression (Figure 2B).

Whereas two identified genes (i.e. *YLL030C*, *YLR241W*) have not been functionally characterized, the other nine genes are involved in a variety of cellular processes (see the *Saccharomyces *Genome Database yeastgenome.org and the YPD database ). *AAD10 *encodes an aryl alcohol dehydrogenase important for amino acid catabolism. Mss11 and Rfx1 are transcription factors involved in the regulation of invasive growth and in DNA damage responses, respectively. Ppg1 has protein phosphatase activity and is involved in glycogen accumulation. Systematic deletion studies suggest that Kre22 (Ydr433w) is involved in glycogen accumulation [38], mitochondrial distribution and morphology [39], and is important in resistance to DNA damaging agents (e.g. hydroxyurea, UV and ionizing radiation) [40]. Vph1 is a subunit of the vacuolar H(+)-ATPase essential for vacuolar acidification. Ste4 functions as the β subunit of the G protein that mediates pheromone-induced signal transduction. *DTR1 *encodes the dityrosine transporter involved in the maturation of the spore wall. It is worth noting that overexpression of Spc98, a component of the spindle pole body [41], causes more severe growth retardation in *rad23Δ dsk2Δ *mutant cells than in wild-type cells (Figure 2B), consistent with previous findings that suggest a role of Rad23 and Dsk2 in spindle function [19].

### The degradation of Kre22 and YLL030C requires Rad23/Dsk2

**Figure 3:**
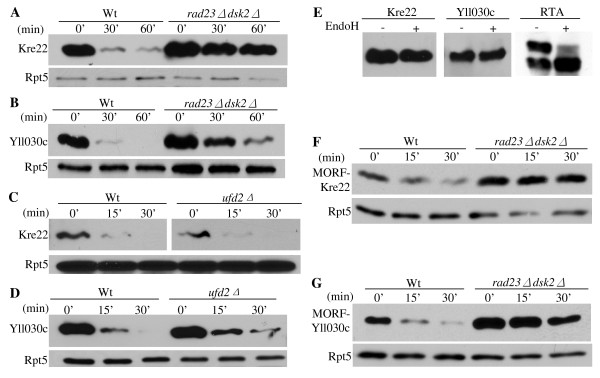
**Kre22 and YLL030C are degraded by a Rad23/Dsk2-dependent pathway**. **(A-B) **Degradation of Kre22 and YLL030C is impaired in cells lacking *RAD23 *and *DSK2*. Wild-type (Y8835) and mutant cells containing a *GAL1 *promoter regulated GST and His6 tagged Kre22 or YLL030C were first grown in raffinose-containing media. Protein expression was then induced by the addition of galactose. Samples were taken after expression shutoff at intervals, processed for immunoprecipitation with GST beads, and analyzed by Western blotting using anti-His6 antibody. Equal amounts of protein extracts were used and confirmed by blotting with anti-Rpt5 antibody in all of the expression shutoff experiments (lower panels). **(C-D) **Degradation of Kre22 and YLL030C is Ufd2-independent. The stability of Kre22 or YLL030C in wild-type or *ufd2Δ *mutant cells was determined by an expression shut-off assay as described in *A*. **(E) **Kre22 and YLL030C are not glycosylated. GST-tagged Kre22 or YLL030C was expressed in wild-type cells and recovered by immunoprecipitation. The immunoprecipitates were mock treated (-) or digested (+) with Endoglycosidase H (Endo H), resolved by SDS-PAGE, and visualized by immunoblotting. If the proteins are N-glycosylated, the bands are expected to migrate faster on the gel after Endo H treatment. A positive control for EndoH treatment of RTA is also included on the right [26]. **(F-G) **Degradation of Kre22 and YLL030C appended with other epitope tags is compromised in cells lacking *RAD23 *and *DSK2*. The stability of Kre22 or YLL030C tagged with C-terminal Ha epitope and the IgG-binding domain from protein A in wild-type and *rad23Δ dsk2Δ *mutant cells was determined as described in *A*, except the immunoprecipitation was done with IgG sepharose and the blots were probed with anti-Ha antibody.

As reasoned above, accumulation of a large amount of substrates can lead to toxicity in cells defective in proteolysis (Figure 1A). We examined the stability of the proteins encoded by the eleven isolated genes in wild-type or mutant cells by an expression shut-off assay. Two proteins, Kre22 and YLL030C are rapidly degraded in wild-type cells, but significantly stabilized in cells lacking *RAD23 *and *DSK2 *(Figure 3A, B), suggesting that they are degraded by the Rad23/Dsk2 pathway. Deletion of *RAD23 *and *DSK2 *does not significantly alter the half-life of the other nine proteins identified by our screen (data not shown).

Previously, we demonstrated that Rad23 works with two different co-factors Ufd2 (a Ub chain elongation factor, E4) and Png1 (a deglycosylation enzyme) to degrade distinct substrates [20,26]. Here, we examined whether Kre22 or YLL030C turn-over requires Ufd2 or Png1. Deletion of Ufd2 does not alter the degradation of Kre22 or YLL030C (Figure 3C, D). The Png1-Rad23 complex is involved in degrading glycosylated proteins [26]. We found that neither protein is glycosylated (Figure 3E), suggesting that Kre22 and YLL030C are not Png1 substrates. It is likely that these substrates would require co-factors other than Ufd2 and Png1 for their degradation.

Since these substrates are tagged with GST moiety at their N-terminus, one concern of our results is that the degradation of these proteins may be altered by the GST fusion. To this end, we obtained the MORF plasmids expressing YLL030C and Kre22, which are tagged at their carboxyl-terminus with both His6 and the IgG-binding site from protein A (Open Biosystems) that theoretically could also affect protein degradation. As shown in Figure 3, YLL030C and Kre22 are still degraded in Rad23/Dsk2-dependent manner (Figure 3F, G), suggesting that the involvements of Rad23 and Dsk2 in Kre22 and YLL030C degradation are not due to artifacts caused by GST tags. Overexpression of these MORF plasmids also led to growth retardation in *rad23Δ dsk2Δ *mutant cells (data not shown).

### Kre22 and YLL030C are ubiquitylated, proteasomal substrates

**Figure 4:**
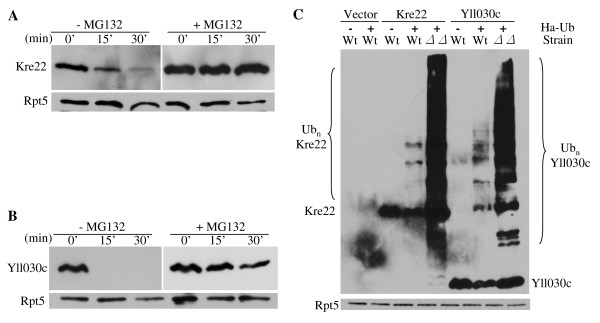
**Ubiquitin and the proteasome are involved in Kre22 and YLL030C degradation**. **(A-B) **Kre22 and YLL030C are degraded by the proteasome. Wild-type yeast cells expressing Kre22 or YLL030C were treated with or without the proteasome inhibitor MG132. To facilitate the uptake of MG132 by wild-type yeast cells, we used L-proline as the nitrogen source in the growth medium and added a small amount of SDS (0.003%). **(C) **Kre22 and YLL030C are ubiquitylated. GST- and His6-tagged substrates were co-transformed with Ha-tagged Ub into wild-type or *rad23 dsk2 *mutant cells. Kre22 or YLL030C was precipitated with GST beads and analyzed by immunoblotting first with anti-Ha antibody and later anti-His6 antibody. Ubiquitylated and non-ubiquitylated Kre22 and YLL030C proteins are indicated on two sides of the upper panel. Rpt5 (bottom panel) is used as a loading control.

Are these newly identified Rad23/Dsk2 substrates destroyed by the proteasome or some other proteolytic activity (e.g. lysosome)? To assess the involvement of the proteasome in the degradation of Kre22 or YLL030C, we carried out a simple proteasome inhibition assay [31]. We found that the degradation of Kre22 or YLL030C is impaired upon MG132 treatment (Figure 4A, B), suggesting that these proteins are degraded by the proteasome.

Most, but not all, proteasomal substrates are ubiquitylated. The function of Rad23/Dsk2 in proteolysis requires their UBA domains, which bind to Ub [4]. To determine whether the newly identified substrates are ubiquitylated, we co-transformed the plasmid expressing the GST- and His6-tagged substrate with the plasmid bearing Ha-tagged Ub into wild-type or *rad23Δ dsk2Δ *mutant cells. GST-tagged substrates were immunoprecipitated from cellular lysates with GST beads, and processed for Western blotting with anti-Ha antibody to detect ubiquitylated substrate. We found that both Kre22 and YLL030C are ubiquitylated (Figure 4C). Consistent with the function of Rad23 and Dsk2 at a pre-proteasome but post-ubiquitylation step [4,5], much more ubiquitylated species were detected in the *rad23Δ dsk2Δ *mutants than in wild type cells (Figure 4C).

### The involvement of specific Ub receptor for the degradation of Kre22 or YLL030C

**Figure 5:**
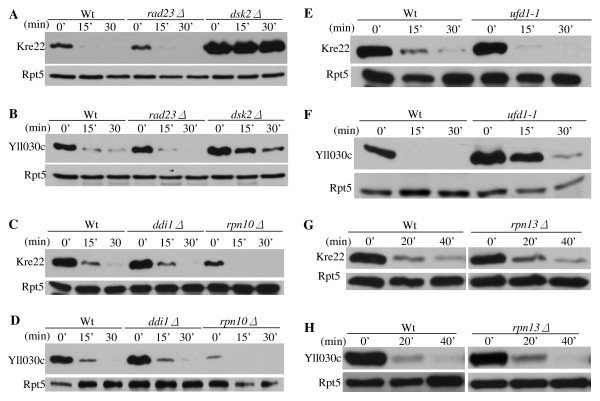
**Kre22 and YLL030C degradation in yeast cells lacking individual Ub receptor**. **(A-H) **Degradation of GST-tagged Kre22 and YLL030C in cells lacking *RAD23, DSK2, DDI1, RPN10, RPN13, UFD1 *was determined as described in Figure 3A.

Ub-binding proteins that function in proteasome-mediated proteolysis include three UBA/UBL proteins (i.e. Rad23, Dsk2, Ddi1), two proteasome-associated factors Rpn10 and Rpn13, and the Ufd1-Cdc48-Npl4 complex [2,21]. These Ub-receptors have different substrates and sometimes carry out overlapping functions [2]. We examined the stability of GST-tagged Kre22 and YLL030C in yeast cells lacking individual Ub receptor (Figure 5A-H). Both Dsk2 and Ufd1 are required for efficient degradation of YLL030C (Figure 5B, D, F, H). Interestingly, Dsk2 is the only Ub receptor involved in Kre22 degradation (Figure 5A, C, E, G). Furthermore, consistent with the restriction of Dsk2 by Rpn10 [42], Kre22 and YLL030C are degraded faster in *rpn10Δ *cells than in wild-type cells (Figure 5C, D).

### Kre22 overexpression suppresses Dsk2-induced toxicity

**Figure 6:**
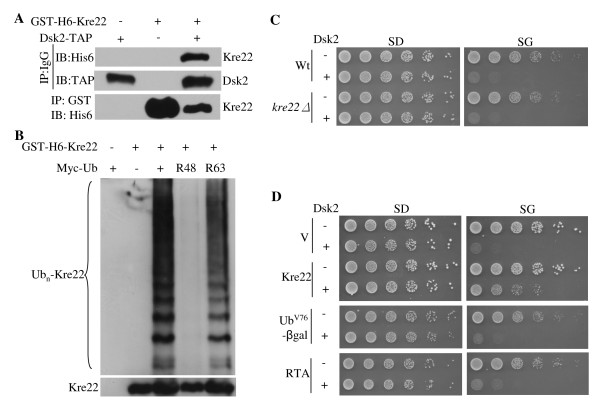
**The interactions between Kre22 and Dsk2**. **(A) **Kre22 binds Dsk2. Co-immunoprecipitation analysis of the interaction between GST-His6-tagged Kre22 and Dsk2-TAP was done as previously described [20]. Briefly, proteins were extracted from cells expressing endogenous Dsk2-TAP and galactose-inducible GST-His6-tagged Kre22 and immunoprecipitated with either IgG or GST-beads as indicated on the left. Immunoprecipitates were resolved by SDS-PAGE and probed with indicated antibodies. The identity of the bands is indicated on the right. The antibodies for IP and immuno-blot (IB) are shown to the left of the panels. **(B) **Kre22 ubiquitylation involves Lys48-linked Ub chains. GST-His6-tagged Kre22 was co-transformed with myc-tagged wild-type or mutant Ub into wild-type cells. Kre22 was precipitated with GST beads and analyzed by immunoblotting first with anti-myc antibody and later anti-His6 antibody. Ubiquitylated and non-ubiquitylated Kre22 proteins are indicated on the left of the panels. **(C) **The deletion of *KRE22 *does not alter *DSK2*-induced toxicity. The spotting assay was done as described in Figure 1A. The vector plasmid or plasmid bearing *DSK2 *regulated by the galactose-inducible *GAL1 *promoter was separately introduced into wild-type or *kre22Δ *cells as indicated. Exponentially growing yeast cells were spotted onto glucose-containing SD-ura (expression off) and galactose-containing SG-ura (expression on) media in serial five-fold dilutions. The strains used and the plasmid for DSK2 overexpression are labeled on the left of the panels. **(D) **overexpression of *KRE22*, but not two other proteasomal substrates RTA and Ub^V76^-V-βgal, suppressed *DSK2*-trigged slow growth in wild-type cells. The spotting assay was done as described above. The expression of these substrates is also regulated by the *GAL1 *promoter. The identities of these substrates and the plasmid for *DSK2 *overexpression are listed to the left of the panels.

To our knowledge, Kre22 is the first substrate that requires only Dsk2 but none of the other Ub receptors for its degradation (Figure 5A, C, E, G). Supporting the direct link between Dsk2 and Kre22, we detected the Dsk2-Kre22 interaction by co-immunoprecipitations (Figure 6A). It will be important to determine the specific domains of Dsk2 involved in Kre22-binding and degradation, and the role of ubiquitylation in the Dsk2-Kre22 interaction. Since little is known about Dsk2-specific substrates, we examined the Ub linkage assembled onto Kre22. Lys48- and Lys63-linked chains were recently linked to the Dsk2 pathway [42]. Specifically, we employed Ub mutants (i.e. Lys48Arg, Lys63Arg) that had either Lys48 or Lys63 replaced with Arg, which in turn blocked Ub conjugation to these lysine residues [2,3,6]. We found that Kre22 ubiquitylation was abolished by Lys48Arg mutation (Figure 6B), suggesting that the Ub chain attached to Kre22 contained Lys48 linkage.

Among Ub-binding proteins involved in proteasome-mediated proteolysis, Dsk2 has the unique property that its overexpression caused toxicity [19,42]. We examined whether the deletion or overexpression of Kre22 could counteract overdose of Dsk2 (Figure 6C, D). Interestingly, increased expression of Kre22 partially suppressed Dsk2-induced toxicity (Figure 6D). We then asked whether other proteasomal substrates could keep Dsk2 in check. We employed RTA, a Rad23, but not Dsk2 substrate, and Ub^V76^-Val-βgal that is regulated by both Rad23 and Dsk2. Interestingly, the suppression of Dsk2 is substrate specific since the overexpression of RTA or Ub^V76^-Val-βgal could not overcome Dsk2-triggered toxicity (Figure 6D). YLL030C overexpression also partially suppressed Dsk2-induced growth retardation (data not shown)

## Discussion

We describe herein the use of a large-scale SDL screen as a new approach to identify proteolytic targets of the Rad23/Dsk2-dependent pathway. Although many strategies have been developed for substrate isolation, the identification of physiological targets of proteolytic pathways has proven to be quite challenging. For example, the first genetically defined Ub-dependent proteolytic pathway (i.e. N-end rule) was discovered in the mid-1980s [37,43]. Despite over a decade of intense efforts, the first cellular substrate of the N-end rule pathway was identified fortuitously. A key cell cycle protein, Scc1 is cleaved at the metaphase-anaphase transition [44] and the resulting carboxyl-terminal fragment is destroyed by the N-end rule pathway to maintain chromosome stability [24]. Interestingly, overexpression of Scc1 leads to lethality in mutant cells lacking the N-end rule E3 enzyme (Figure 1A, and reference [24]). This finding highlights the difficulty in obtaining proteolytic substrates and also suggests that an SDL screen could be effective for substrate identification.

An SDL approach was first employed to uncover genetic interactions in DNA replication or chromosome segregation [32,33]. More recently, it was demonstrated that SDL screens could be used to identify novel targets of a protein kinase (i.e. Pho85) [28]. Our study further extends the effectiveness of the SDL screen to the Ub/proteasome system. The advantages of SDL as a screening tool for proteolytic substrate identification include that the assay is performed *in vivo*, and the protein expression levels are enhanced by the strong promoter (i.e. *GAL1*). Like other approaches for target discovery, SDL screens have limitations. Of approximately 5000 yeast genes we screened, only two Dsk2 substrates were isolated. Several factors may contribute to the high false negative level: 1) redundancy of various substrate delivery pathways [7,8,23]. Several known Dsk2/Rad23 substrates (e.g. Sic1) [7,8,23] were not recovered in this screen. In the absence of Dsk2/Rad23, other Ub adaptor molecules (e.g. Rpn10, Cdc48, Ddi1, Rpn13) may maintain sufficient proteolysis [7,8]; 2) genes that lead to severe growth defects in wild-type cells with increased dosage are likely to be excluded from such analysis; 3) not all substrates would elicit enhanced fitness defects in proteolysis mutants (Figure 1); 4) the screen is an indirect way for target discovery. Large number of false positives, partly due to a low colony size cutoff, may mask some true positives; 5) screening strategy and criteria for target identification may need further refinement. For example, the rapid turnover or absence of particular substrates might be a requirement under very specific environmental or physiological conditions, which were not covered by our standardized screening protocol (e.g. degradation of nuclear-specific Far1 in response to mating pheromone). Nevertheless, given the difficulty involved in substrate isolation, we believe that SDL provides another way to delineate the functional relationship between a substrate and the specific degradation pathway.

To our knowledge, GST-Kre22 is the first substrate that requires only Dsk2, but not other Ub receptors (e.g. Rpn10, Rad23) for degradation (Figure 5A, C, E, G). Interestingly, overexpressed Kre22 but not other proteasomal substrates suppresses Dsk2-induced toxicity (Figure 6D). Although the precise mechanism underlying the Dsk2-triggered toxic effect is not clear [42], one possible explanation is that excessive Dsk2 may engage in the abnormal activities that interfere with cell growth. And increased amount of specific substrates may keep Dsk2 in check. Our results suggest that Kre22 could serve as an important tool to understand the Dsk2-dependent proteolysis.

Most proteins identified are not direct proteolytic substrates of Rad23 and Dsk2. This is not unexpected since other genes that function in the linear pathway of Rad23/Dsk2 target may also induce toxicity in *rad23 dsk2 *mutant cells upon their overexpression. For example, in a separate study on the function of Rad23 and Dsk2, we identified a cell cycle protein involved in the spindle pole body (SPB) duplication as a degradation target of Rad23 and Dsk2 (C.L., H.R., unpublished results). Interestingly, this protein is known to act upstream of Spc98, a component of the SPB [41]. It is likely that increased levels of Spc98 mimic the accumulation of this Rad23/Dsk2 target involved in spindle regulation to affect SPB duplication, which in turn triggers growth retardation in *rad23 dsk2 *mutant cells. Therefore, although the other eight proteins identified by our screen are not regulated directly by Rad23 and Dsk2, we suspect that these proteins work with Rad23/Dsk2 targets *in vivo *and cellular responses to their overexpression require the function of Rad23 and Dsk2 (Figure 2B).

## Conclusion

In conclusion, as proof-of-concept, we show that an SDL screen offers an effective, complementary method to elucidating biological functions of proteolytic pathways. Dsk2 was first identified 11 years ago in a genetic screen for genes involved in SPB duplication [19]. To our knowledge, the Spc98-induced toxicity in *rad23 dsk2 *mutant cells is the second piece of evidence linking Dsk2 and Rad23 to SPB duplication, which will help us to unravel the specific role of Rad23 and Dsk2 in this process. It is also conceivable that the SDL screen can be adapted to identify relevant ubiquitylation enzymes involved in substrate turnover. The creation of RNAi knock-down libraries and ORF overexpression collections would extend the use of SDL screens in the Ub/proteasome system to higher eukaryotes.

## Abbreviations

SPB: spindle pole body; Ub: ubiquitin; UBA: ubiquitin-associated; UBL: ubiquitin-like; SDL: synthetic dosage lethality; His6: hexahistidine; GST: glutathione Stransferase.

## Authors' contributions

CL, DvD and YL performed the experiments. BA and HR conceived the study and designed the experiments. CL, DvD, BA and HR wrote the manuscript.
